# Path loss characterization of a compact dual-band mmWave antenna in urban microenvironments

**DOI:** 10.1371/journal.pone.0352862

**Published:** 2026-07-02

**Authors:** Praveen Kumar, G. D. Goutham Simha

**Affiliations:** Manipal Institute of Technology, Manipal Academy of Higher Education, Manipal, India; Model Institute of Engineering and Technology, INDIA

## Abstract

This work presents a compact dual-band millimeter wave (mmWave) antenna that operates at 17–27 GHz and 36–43 GHz, with resonance frequencies centred at 21.1 GHz and 38.3 GHz, respectively. The multiple embedded arc-shaped mmWave antenna geometry provides compact, effective dual-band operation. The concentric arcs of the antenna structure are effectively optimized to produce a dual band of operation with good impedance matching. The resulting antenna has gain of 4.8 dBi at 26 GHz and 6.1 dBi at 37 GHz frequencies. The developed antenna is intended for widespread usage in satellite and 5G networks. Despite providing significant bandwidth for 5G and other next-generation communication systems, mmWave frequencies are susceptible to path loss, particularly in congested urban areas. This study further investigates the performance of the designed dual-band mmWave antennas in various urban environments. Both Line-of-Sight (LOS) and Non-Line-of-Sight (NLOS) scenarios are considered to capture realistic deployment conditions. To accurately model propagation behaviour, the analysis incorporates the ABG and CI path-loss models, which comprehensively account for environmental factors such as shadowing, diffraction, and precipitation.

## 1 Introduction

While more established wireless cellular systems continue to be deployed, the capacity and performance provided by current 4G technologies are becoming inadequate, leading to the proliferation of new wireless systems such as 5G, 6G, and even future communications [1]. Such activities have led to the consideration of millimeter wave (mmWave) frequency ranges (30–300 GHz) in order to meet the needs for larger data speeds, increased capacity, and extremely low latency. These high-frequency bands offer more than adequate capacity to support a variety of applications, including improved mobile broadband, large machine-type communications, and ultra-reliable low-latency communications [2,3]. Despite being subject to weather conditions and non-line-of-sight propagation, the 28 GHz and 38 GHz bands are recognised for 5G mmWave transmission due to their ability to send mmWave frequency transmissions [4,5]. Nonetheless, developing mmWave systems has specific challenges, including significant path loss, system downsizing concerns, and the necessity for beam steering. This decrease in performance boosts the necessity for new types of antennas with superior radiation characteristics in order to minimize losses [6,7]. The ability to manufacture such antennas is critical because future wireless networks that support augmented and virtual reality, V2X communications, and smart cities will require compact, efficient systems [8].

Different methods for designing the millimetre-wave antenna are illustrated in the literature. An E-shaped antenna with H-shaped slots is proposed in [9], which is frequency optimized at mmWave. The Dual Band Antenna is designed to be used for 5G mmWave communication needs. The antenna supports dual-band operation, typically within the 24–28 GHz and 37–40 GHz bands, which are essential for 5G utilization. The dual-band operation facilitates the 5G allocation of mmWave channels. E-shaped patch and H-shaped slots increase the effectiveness of the device’s impedance and radiation characteristics. The compactness of the design is essential for the integration of the antenna into the latest 5G devices. Several rings are stacked and linked to the feedline on the RT duroid 5880 substrate to investigate the operation in the broadband frequency range, as seen in [10]. A metamaterial-based mmWave antenna was used in [11], which is developed to bolster gain and radiation features, in the 5G frequency spectrum. The antenna is intended for use in the mmWave spectrum especially 24–40 GHz. To create a broadband mmWave antenna, [12] incorporates traditional circular and rectangular antennas with circular slots. The dual band antenna in [5] uses elliptical slots to achieve better compactness and performance of the antenna in mmWave frequencies. An arc-shaped reduced ground plane simple antenna design provides an operational frequency of 25.8–30.2 GHz [13]. A rectangular patch antenna is altered by carving the edges of the radiating elements and a rectangular slot onto the ground plane in [14]. The designed antenna operates in two bands in the frequency spectrum of 26.3–28.8 GHz and 34–41.5 GHz. A dual band frequency operation of 26.7–30.3 GHz, 35.8–41.2 GHz antenna is designed using the hexagonal ring and split ring as radiating element with the modified ground plane in [15]. The design of mmWave antennas is challenging as they are primarily associated with high frequencies and small wavelengths. Downsizing the antenna with such frequencies by maintaining good impedance matching and radiation properties is difficult. The size restrictions themselves are also a problem when it comes to patch antennas, as many applications demand wider impedance bandwidth. Finally, the influence of environmental conditions such as shadowing, absorption, and rain also has to be taken into consideration.

The design of a compact dual band mmWave antenna that operates in the 17–27 GHz and 36–43 GHz frequency ranges is demonstrated in this study. The antenna is made on a Rogers RT duroid 5880 substrate and has an overall size of 7 x 7 × 0.5 mm^3^. The broader impedance bandwidth in dual bands is made possible by the many arcs that the semi-concentric arcs form onto the radiating element and decreased ground plane. The designed antenna exhibits good gain and stable radiation properties. The remainder of the article is organized as follows: the design methodology of the antenna is described in Sect 2. Sect 3 depicts the outcome of the designed antenna and validation through prototyping. A comprehensive path loss analysis of the designed antenna is performed in Sect 4. Concluding observations are offered in the final section.

## 2 Antenna design

**Fig 1:**
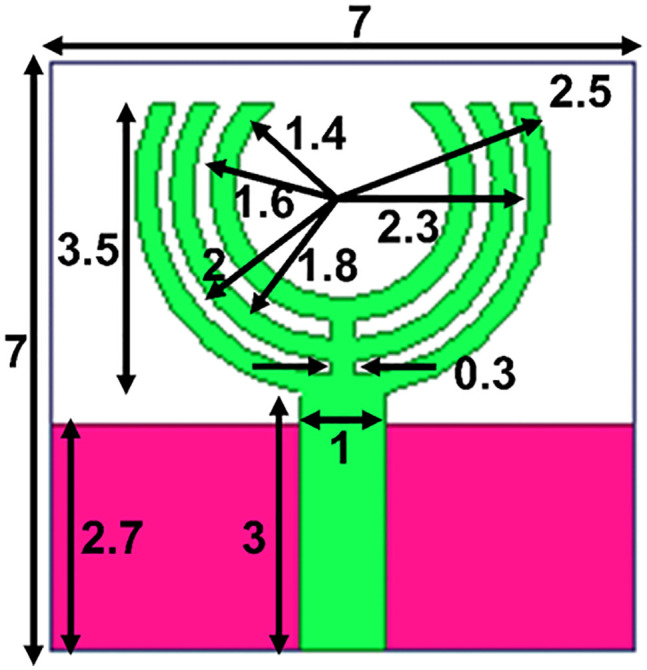
The physical dimension of the designed antenna (mm).


The concentric arc mmWave antenna is composed of many concentric arcs paired with a feed structure and lowered ground plane for optimal power delivery. The design efficiently contributes to greater radiation properties and better resonance by utilizing the notion of numerous radiating routes. The antenna offers better impedance bandwidth and gain by carefully adjusting the radius, curvature, and spacing of these arcs. The designed antenna has an overall dimension of 7 × 7 × 0.5 mm^3^ on Rogers RT duroid 5880 substrate, as depicted in Fig 1. Eqs (1)–(2) are used to compute the radius of a standard circular patch antenna in the first stage [16]. Eqs (1) and (2) are used to estimate initial patch dimensions, although resonance is only achieved after further structural modifications as shown in the antenna evolution. The proposed antenna has resonances at 21.1 GHz and 38.3 GHz, respectively, and works in the dual frequency range of 17–27 GHz and 36–43 GHz.

$$\begin{array}{c} R_{p}=\frac{F_{O}}{{\left\{\text{1}+\left(\frac{\text{2}H}{\pi \text{K}F_{O}}\right)\left[ln\left(\frac{\pi F_{O}}{\text{2}H}\right)+\text{1}.\text{7}\text{7}\text{2}\text{6}\right]\right\}}^{\frac{\text{1}}{\text{2}}}} \end{array}$$ (1)

where $F_{O}$ is


$$\begin{array}{c} F_{O}=\frac{\text{8}.\text{7}\text{9}\text{1}\times {\text{1}\text{0}}^{\text{9}}}{f\text{K}} \end{array}$$

$$\begin{array}{c} {Effective\;R}_{p}=R_{p}\;{\left\{\text{1}+\left(\frac{\text{2}H}{\pi R_{p}\text{K}}\right)\left[ln\left(\frac{\pi R_{p}}{\text{2}H}+\text{1}.\text{7}\text{7}\text{2}\text{6}\right)\right]\right\}}^{\frac{\text{1}}{\text{2}}} \end{array}$$ (2)

*f* is a resonant frequency of 38 GHz, *K* is a dielectric constant of 2.2, and the thickness of the substrate *H* is 0.5 mm. Putting these values in Eqs (1)–(2) results in $R_{p}$ is 1.4 mm, and the effective radius of the patch is 1.6 mm.

**Fig 2:**
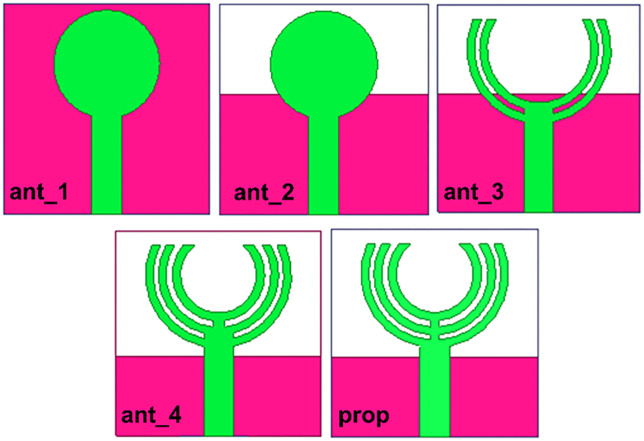
The geometric structural creation phases of the proposed antenna.

**Fig 3:**
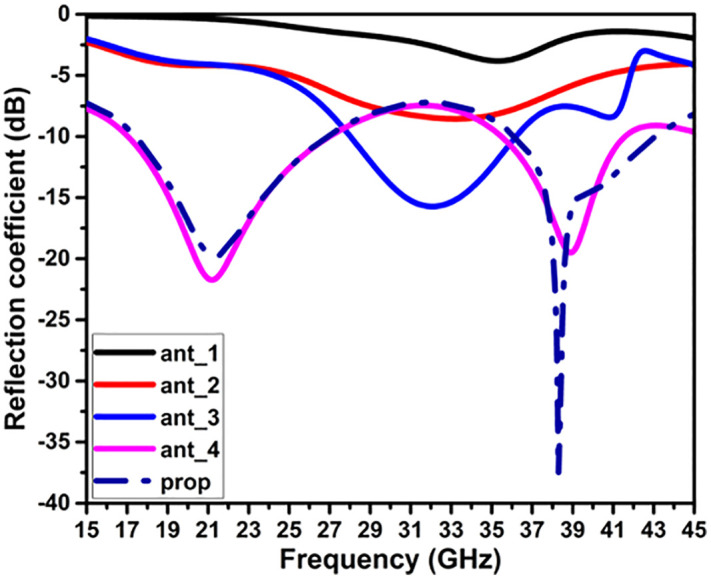
The geometric structural creation phases of the proposed antenna.

Fig 2 illustrates the construction of the circular patch antenna (ant_1) with the whole ground plane and an effective radius of 1.6 mm. As a result, the reflection coefficient (S11) is not below −10 dB, as shown in Fig 3, indicating that the impedance of ant_1 is more reactive than resistive. As seen in Fig 2, in step 2, the antenna’s (ant_2) ground plane is shrunk from 7 mm to 4 mm. The uniform current distribution is impacted by a shorter ground plane, and improved impedance bandwidth is achieved. Similar modifications are made to the antenna’s radiator in stages 3, 4, and 5, which yields a oncentric arc radiator with a ground plane of 2.7 mm for the suggested antenna. These modifications are made utilizing the circular and rectangular slots. The proposed antenna evolution and related S11 curves are presented in Figs 2 and 3 correspondingly.

### 2.1 Parametric analysis

**Fig 4:**
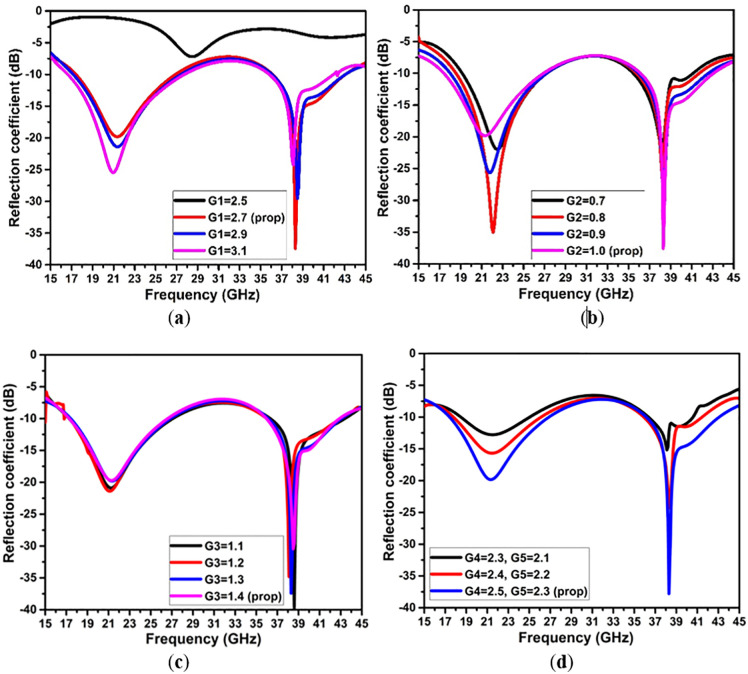
The parametric analysis of the designed antenna (a) G1, (b) G2, (c) G3, and (d) G4 and G5.

The proposed dual band mmWave antenna is subjected to parametric analysis in order to examine the effects of changes to the antenna’s physical dimensions, including the ground plane (G1), feedline width (G2), and patch alterations encompassing the inner circle (G3) and outermost circles (G4, G5). As shown in Fig 4(a), the change of G1 is performed in a linear step of 0.2 mm from 2.5 mm to 3.5 mm. With proper impedance matching, the G1 value of 2.7 mm offers a significant bandwidth at the lower frequency side in addition to a broader impedance bandwidth at the higher frequency side. Improvements to the ground plane can assist in achieving improved impedance matching and may have an impact on lumped components. The G2 is then varied between 0.7 and 1.3 mm. The G2 values of 0.7 and 0.8 mm show resonance shifting to the lower frequency side, while larger values cause the impedance matching to be disturbed and shift the higher resonance to the higher frequency side, as depicted in Fig 4(b). Similar to this, Fig 4(c) and 4(d) shows how the patch’s structure is altered in G3 and G4 in accordance with the G5. A minor increase in bandwidth at the higher frequency is shown by the increased value of G3 from 1.1 to 1.4 mm. G4 and G5, the outermost concentric arcs, have different synchronization at 2.5 mm and 2.3 mm, respectively, offering an optimal bandwidth between 17 and 27 GHz, resonating at 21.1 GHz and 36 and 43 GHz, resonating at 38.3 GHz.

## 3 Results and discussions

**Fig 5:**
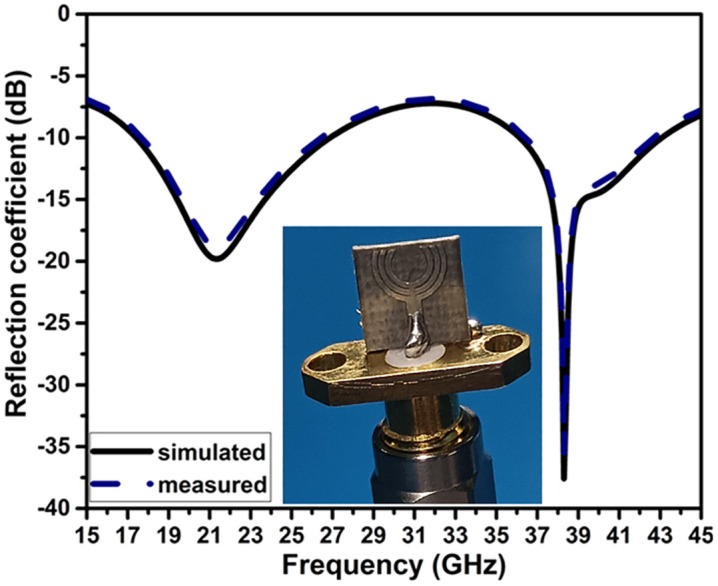
The S-parameter curve of the proposed mmWave antenna.

This section describes the radiation characteristics and reflection coefficient curve of the dual band mmWave antenna that was constructed. The proposed antenna’s observed and simulated reflection coefficient curve is shown in Fig 5. As shown in Fig 5, it displays the antenna’s operational frequency between 17–27 GHz and 36–43 GHz. The concentric arcs are etched into the patch to create the dual band, and the ground plane is dropped to achieve broad bandwidth impedance matching.

**Fig 6:**
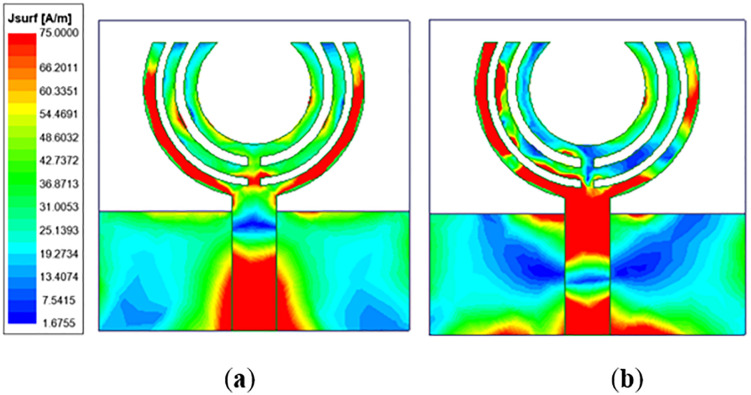
Surface current distribution plot of the antenna (a) 21.1 GHz, and (b) 38.3 GHz.

Fig 6(a)–6(b) displays the current distribution on the ground plane and radiating plane at the resonance frequencies of 38.3 GHz and 21.1 GHz. The patch’s center, feedline, and ground plane are where the most current is focused.

**Fig 7:**
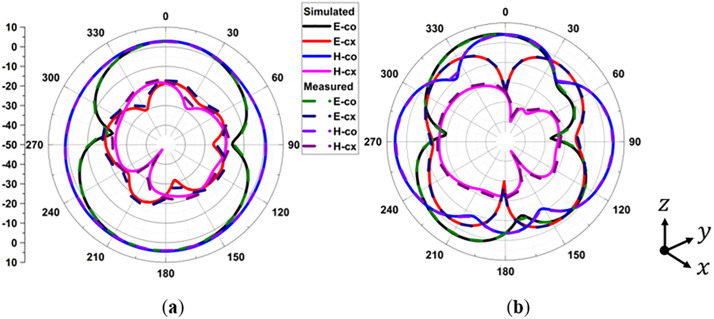
The radiation patterns of the proposed antenna at resonance frequencies: (a) 21.1 GHz and (b) 38.3 GHz.

**Fig 8:**
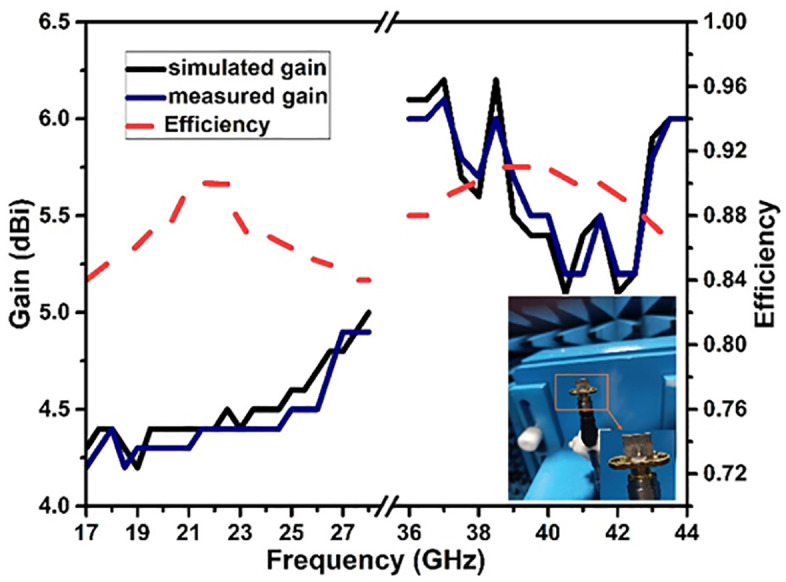
The gain, efficiency vs frequency curve of the designed antenna.

In the anechoic chamber, the designed antenna’s radiation properties are evaluated at the resonance frequencies of 21.1 GHz and 38.3 GHz, as depicted in Fig 7(a)–7(b). The co- and cross-polarization at the two principal planes, XZ and YZ, depict bidirectional and omnidirectional patterns, respectively. The gain of the proposed antenna is plotted against frequency in the two operating bands shown in Fig 8. The maximum gain is 4.8 dBi and 6.1 dBi at the frequencies of 26 GHz and 37 GHz, respectively, in both bands. The efficiency of the antenna in both bands are greater than 0.83 as depicted in Fig 8.

### 3.1. Comparative analysis

Table 1 presents a comparative analysis of a developed dual band mmWave antenna and comparable existing antennas, taking into account many factors like dimensions, bandwidth, and gain. The presented antenna offers a balance of gain (4.8 and 6.1 dBi), compactness, and dual-band operation, which is critical for multi-band 5G applications in space-constrained devices in contrast to the existing antennas.

**Table 1:** The performance of proposed antenna in concordance with the existing antenna.

Ref	Antenna Dimensions (mm^3^)	Technique	Number of bands	Impedance Bandwidth (GHz)	Fractional Bandwidth (%)	Gain (dBi)
[14]	20 × 20 × 0.7	Rectangular patch with parasitic elements	2	26.3–28.8, 34–41.5	9.17, 19.86	–
[15]	6 × 8 × 0.254	Metamaterial	2	26.7–3 0.3, 35.8–41.2	12.63, 14.02	4.9, 3.5
[17]	7 × 4.5 × 1.5	Modified elliptical radiator and the ground plane	1	40 - 80	66.67	6.7
[18]	15 × 15 × 0.203	Modified circular radiator with slotted ground plane	2	25.5 - 30.5, 35.5 −40	17.85, 11.92	5.7, 6.9
[19]	8 × 10 × 0.254	Square Frame T radiator	1	25.2 - 37	37.94	–
[20]	10 × 10 × 0.25	T shape radiator with bent strip	2	24–39.5, 44.4–50.8	48.81, 13.44	–
[21]	8.2 × 12.2 × 0.4	Fractal	1	27.5 - 28.6	3.92	–
[22]	13.4 × 13.4 × 0.787	Meander line	1	17.5-40	78.2	6
[23]	5 × 6.8 × 1.524	Coplanar patch	1	23-30.5	28.03	**–**
Proposed	7 × 7 × 0.5	Concentric arcs	2	17-27, 36-43	**45.45, 17.72**	4.8, 6.1

* Not available

## 4 Path loss model

When constructing a wireless communication system, path loss (PL), a crucial characteristic of electromagnetic wave transmission, must be taken into account for system enhancement. The current study utilizes the alpha beta gamma (ABG), close-in (CI) models, Received Signal Strength Indicators (RSSI), Reference Signal Received Power (RSRP), Reference Signal Received Quality (RSRQ) to analyze the PL of the proposed dual band mmWave antenna. The ABG and CI path loss models are frequency-independent, comprehensive frameworks for characterizing large-scale propagation path loss over a wide range of frequencies within a given scenario. The CI model aligns well with the existing 3GPP models with a small modification: it replaces the arbitrary, non-physical constant with a frequency-dependent term that accurately represents free space path loss at a one-meter distance. The following Eqs (3)–(4) describe the ABG and CI models [24].


$$\begin{array}{c} {\text{PL}}^{\text{ABG}}(\text{f},\text{ d})[\text{dB}]=\text{1}\text{0}\alpha {\text{log}}_{\text{10}}\left(\frac{\text{d}}{\text{1m}}\right)+\beta +\text{1}\text{0}\gamma {\text{log}}_{\text{10}}\left(\frac{\text{f}}{\text{1GHz}}\right)+\overset{\text{ABG}}{\underset{\sigma }{\chi }} \end{array}$$ (3)

$$\begin{array}{c} {\text{PL}}^{\text{CI}}(\text{f},\text{ d})[\text{dB}]=\text{FSPL}(\text{f},\text{ 1m})[\text{dB}]+\text{10n}{\text{log}}_{\text{10}}(\text{d})+\overset{\text{CI}}{\underset{\sigma }{\chi }} \end{array}$$ (4)

In this formula, ${\text{PL}}^{\text{ABG}}(\text{f},\text{ d})\;$refers to path loss in decibels over frequency and distance, with coefficients α and γ describing how path loss relates to distance and frequency. The term β represents an optimized offset value, d is the 3D separation between the transmitter and receiver in meters, and $\overset{\text{ABG}}{\underset{\sigma }{\chi }}\;$accounts for large-scale signal fluctuations around the mean. Similarly, $\overset{\text{CI}}{\underset{\sigma }{\chi }}$ refers to large-scale fluctuations around the mean in the CI model.

The path-loss model ABG and CI coefficient models in this section are not derived from field measurements for the proposed antenna configuration, nor were they based solely on a single standardized channel model. They are adapted directly out of [24,25] “Propagation Path Loss Models in 5G Urban Micro- and Macro-Cellular Scenarios, which condensed findings of twenty different measurement campaigns and ray-tracing studies carried out at NYU WIRELESS, Nokia, Aalborg University, Qualcomm, and Aalto University. These studies were conducted in the 2GHz −73.5GHz frequency range and 5m-1429m distance.

The path-loss exponent (PLE) was estimated in the CI model using minimum mean-square error (MMSE) on a combined dataset of measurements and ray-tracing results. The values in Table 1 of the reference [24] are:

UMi Street Canyon LOS: n = 2.0; UMi Street Canyon NLOS: n = 3.1; UMi Open Square LOS: n = 1.9; UMi Open Square NLOS: n = 2.8; UMa LOS: n = 2.0; UMa NLOS: n 2.7.

The LOS PLEs equal about 2.0, which is consistent with the theoretical value of the exponent of a free space; hence, it follows a physical sanity check of the values that have been empirically determined. Nobel frequency dependence is realized using the one-metre free space reference term.

$FSPL(f,\;\text{1}\;m)\;=\;\text{2}\text{0}{\text{log}}_{\text{1}\text{0}}(\text{4}\pi f/c),$ for which no additional fitting is necessary.

In the case of the ABG model, the three parameters (alpha, beta, gamma) were calculated by optimising the MMSE using the closed-form solutions given in the appendix (Eqs 10, 11, and 12 of this reference). The parameters in the UMi Street Canyon NLOS scenario at 2 to 73.5 GHz are 8.0, 3.5, 24.4, 24.4, 1.9, and a shadow-fading standard deviation of 8.0, 0–1 (Table 2). Such coefficients are anchored in multi-institutional empirical data, rather than a single standard such as 3GPP TR 38.901. In this case alone (UMa), more than 186,000 data points were used (Table 4). Any path-loss exceeding 180 dB was then pegged to this value, which aligns with the assumption about the high-gain antenna and receiver sensitivity that formed the basis of the measurement camps.

**Table 2:** The frequency-dependent rain rate calculations from [26].

Frequency	K	α
**21 GHz**	0.0751	1.099
**38 GHz**	0.3081	0.9624

**Table 3:** Specific Attenuation (dB/km) from [26].

Rain Rate	Condition	21 GHz	38 GHz	Difference
**10 mm/hr**	Moderate	0.94	2.82	1.88 dB/km
**15 mm/hr**	Heavy	1.47	4.17	2.7 dB/km
**25 mm/hr**	Dense/Tropical	2.58	6.82	4.24 dB/km

**Table 4:** Measurement over a representative 500 m urban link.

Rain rate	21 GHz	38 GHz
**10 mm/hr**	0.47 dB	1.41 dB
**15 mm/hr**	0.74 dB	2.05 dB
**25 mm/hr**	1.28 dB	3.31 dB

A critical methodological point is that, the dataset explicitly incorporates both empirical field measurements (M) and ray-tracing simulations (R), as clearly indicated in Sun et al. (2016) [24].

For example:

The UMa NLoS dataset at 2 GHz comprises 69,542 data points collected via both measurement and ray-tracing (Nokia/AAU), Ray-tracing data at 10.25 GHz, 28.5 GHz, 39.3 GHz, and 73.5 GHz for UMa (Nokia) contributed 16,000–17,000 data points per frequency. The total UMa NLoS dataset encompasses 186,498 combined data points across all frequencies and distances. The ray-tracing simulations used in these campaigns were conducted using accurate 3D urban building databases of real city environments (including Helsinki, Aalborg, and New York City), which inherently encode the building height, street width, and obstacle density information that the reviewer correctly identifies as necessary for rigorous LoS/NLoS classification.

These databases provide:

Realistic building footprints and heights, Accurate street widths and canyon aspect ratios, Detailed material properties for reflection and diffraction modeling. The LoS and NLoS path loss characteristics presented in Sect 4.6 are derived from the empirically validated dataset of Sun et al. (2016) [24], which consolidates over 186,000 data points from 20 measurement campaigns and ray-tracing studies across 2–73.5 GHz in urban micro- and macro-cellular environments. LoS conditions are defined by the presence of an unobstructed direct path between the transmitter and receiver, whereas NLoS conditions occur when intervening buildings or structures block the direct path. The NLoS data were obtained from both empirical field measurements and ray-tracing simulations conducted using accurate 3D urban building databases that encode realistic building heights, street widths, and obstacle distributions. The CI model path loss exponents of n = 3.1 (UMi SC), n = 2.8 (UMi OS), and n = 2.7 (UMa) for NLoS, alongside shadow fading standard deviations of up to 10.0 dB, were adopted directly from this reference and applied to the link budget analysis in Sect 4.6.

### 4.1 Path loss vs distance using ABG model in UMi scenario

**Fig 9:**
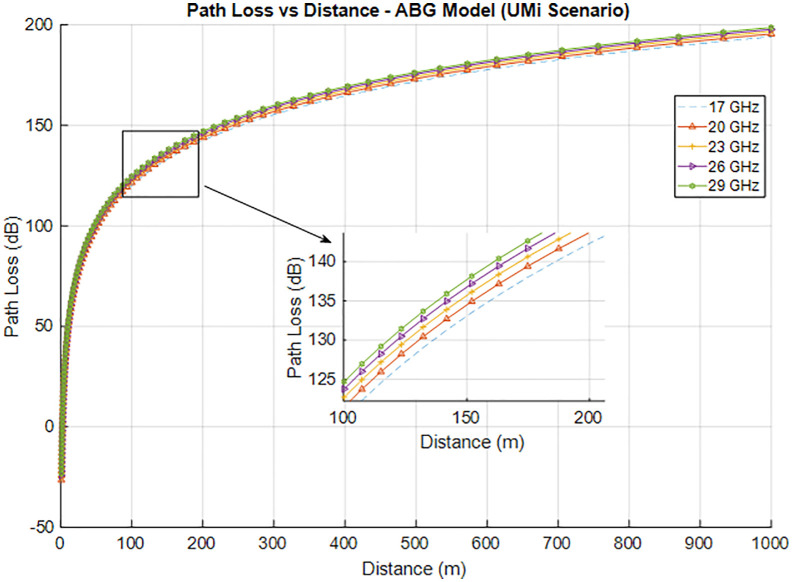
Path Loss vs Distance comparison for ABG Model in UMi Scenario for Band-1 Frequencies.

Fig 9 shows the path loss vs. distance over an ABG model for the proposed dual-band mmWave antenna, which was designed and developed to operate over 17 GHz to 29 GHz as band-1 and 36 GHz to 44 GHz as band-2. A T-R separation distance of up to 1000 meters is considered for the analysis in a typical smart city UMi scenario. Path loss increases exponentially with the distance between the transmitter and the receiver. At 150 meters distance we can see 139dB of path loss is observed for 29GHz, and a 133dB of path loss is recorded for 17GHz signal. Further, a 172 dB path loss is observed at the midpoint between the transmitter and the receiver. A similar trend continues up to 194 dB as the T-R distance is increased to 1000 meters in a smart city UMi scenario at 17 GHz. When the frequency of operation is increased to 29 GHz, the path loss observed is 175 dB at 500 meters which reaches up to 198 dB at a considered distance of 1000 meters.

**Fig 10:**
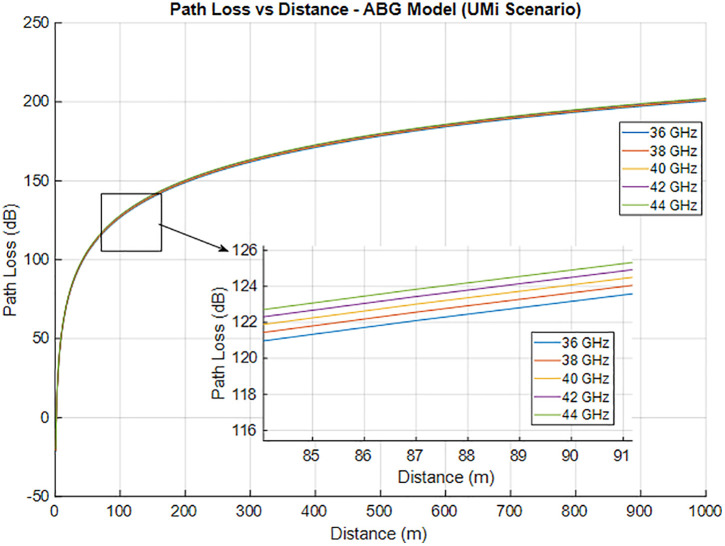
Path Loss vs Distance Comparison for ABG Model in UMi Scenario for Band-2 Frequencies.

By observing Fig 10, one can conclude that a similar path loss trend has been obtained. At around 90-meter distance, we observe a path loss of 122 dB for Band-2 at 36 GHz, which is predominantly high compared to Band-1 at the respective frequency of operation. Furthermore, a marginal 2 dB path loss difference is observed at a 90-meter distance for 44GHz of operation as compared to 36 GHz. At about 500-meter distance a 175 dB path loss is observed for 36GHz of operation. A similar trend follows up to 200 dB when the T-R distance is increased to 1000 meters in a smart city UMi scenario when the operating frequency is 36 GHz. When the frequency of operation is increased to 44 GHz, the path loss increases to 202 dB for a distance of 1000 meters.

### 4.2 Path loss vs distance using CI model in UMi scenario

**Fig 11:**
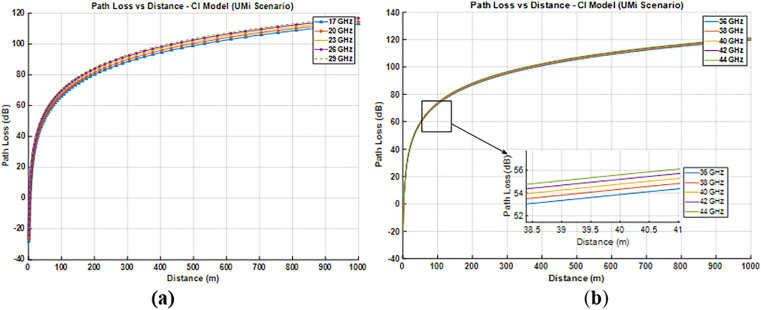
Comparison of Path Loss vs Distance over CI Model in UMi Scenarios for (a) Band-1 and (b) Band-2 frequencies.

Fig 11(a)–11(b) shows the path loss vs distance over a CI model for the dual band mmWave antenna, which is designed and developed to operate over 17 GHz to 29 GHz as Band-1 and 36 GHz to 44 GHz as Band-2. A T-R separation of up to 1000 meters is considered. Path loss increases exponentially with respect to the distance of separation. A 99 dB path loss is observed between the transmitter and receiver at the midpoint. This goes up to 113 dB when the T-R distance is increased to 1000 meters in a UMi when the operating frequency is 17 GHz. When the frequency of operation is increased to 29 GHz, the path loss observed is 104 dB at 500 meters which goes up to 118 dB at a distance of 1000 meters. Similarly for Band-2, 105dB of path loss is observed for 36 GHz at 500 meters distance, and this increases up to 120 dB at 1 km distance. In this system, the CI (Close-in) model performs better than the ABG (Alpha-Beta-Gamma) model, particularly in terms of path loss to distance. The CI model, on the other hand, benefits from basic and realistic free-space reference, which is better suited over large distances in realistic fading scenarios. However, the ABG model has some degree of reliability in that it has multiple parameters (α, β) that are also used in some instances, and some parameters may not work in some places, namely in dense fading and fast fading scenarios. This is one of the reasons why, as distance increases, the ABG parameters are bound to be more prone to path loss model values than the derived case, especially in applications that cross various frequency ranges with diverse environmental attenuation and polarization-mismatch. In the case of MIMO systems, which naturally create spatial diversity and enhance signal strength, the advantages of the CI model, which is low in complexity, ensure better and more reliable coverage even at longer ranges, which concerns on consistency and predictability of the signal.

### 4.3 RSSI at different positions using ABG model in UMi scenario

**Fig 12:**
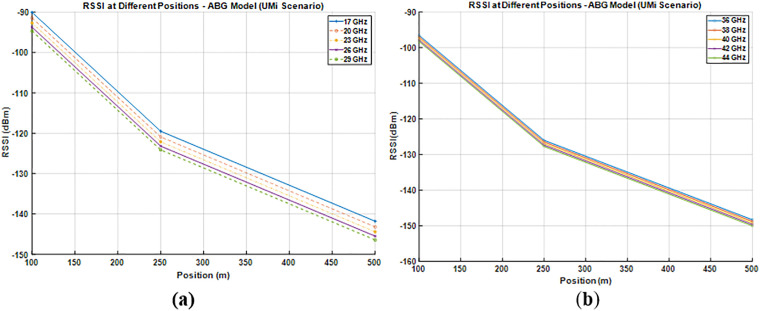
Comparison of RSSI vs Position for ABG Model in UMi Scenario for (a) Band-1 and (b) Band-2 frequencies.

Fig 12(a)–12(b) shows the RSSI measured at different points from the transmitter over an ABG model for the designed antennae operating in Band-1 and Band-2 as mentioned previously. (17 GHz to 29 GHz and 36 GHz to 44 GHz). The received signal strength deteriorates as the receiver moves away from the transmitter. RSSI is −120 dBm at 250 meters, which reduces to −145 dBm at 500 meters for Band-1; a similar trend has been seen with lower RSSI as the distance increases. In 5G systems, due to the usage of mm-wave bands, the correlation between the measurement of RSSI and distance becomes very important, owing to the fact that these bands (Band-1 and Band-2 in our case) are more prone to attenuation with the increase in distance. As the distance between transmitter and receiver increases, the value of RSSI decreases. If the RSSI falls below a threshold value, usually at around −110 to −120 dBm, the signal can no longer be reliably decoded, and the connection is lost. This threshold is crucial in 5G, as the millimeter-wave spectrum used in 5G is highly sensitive to path loss, obstacles, and other environmental factors compared to frequency bands with lower frequencies. Thus, maintaining a strong RSSI will be one of the major challenges for having stable and high-quality communication in 5G networks, and the RSSI versus distance relationship directly impacts network coverage and performance.

### 4.4 RSSI at different positions using CI Model in UMi scenario

**Fig 13:**
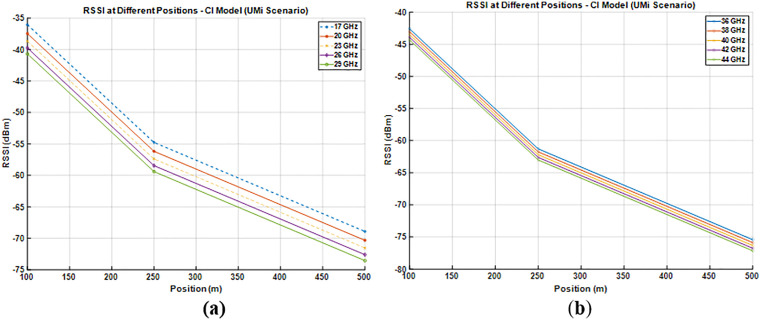
Comparison RSSI vs Position for CI Model in UMi Scenario for (a) Band-1 and (b) Band-2 frequencies.

Fig 13(a)–13(b) shows the measurement of RSSI at different positions from the transmitter over a CI model for the proposed antenna, which is designed and developed to operate over 17 GHz to 29 GHz and 36 GHz to 44 GHz. The received signal strength deteriorates as one moves away from the transmitter. The CI model is better suited for realistic scenarios since RSSI is −55 dBm (17GHz Band-1) at 250 meters distance, which is excellent as per the 5G standards [24,27,28], which reduces to −75 dBm at the midpoint of 500 meters. Even then, the signal strength is very good. Similarly, decreased RSSI is observed in Band-2 as compared to Band-1 at 250 meters distance which is around -63dBm despite that the signal is highly reliable as per the 5G specifications. At about 500 meters the RSSI is decreased to -78dBm (44GHz Band-2) which is good for having proper connectivity from Base station and the Mobile station.

### 4.5 Received power vs distance uisng ABG model and CI model in UMi scenario

**Fig 14:**
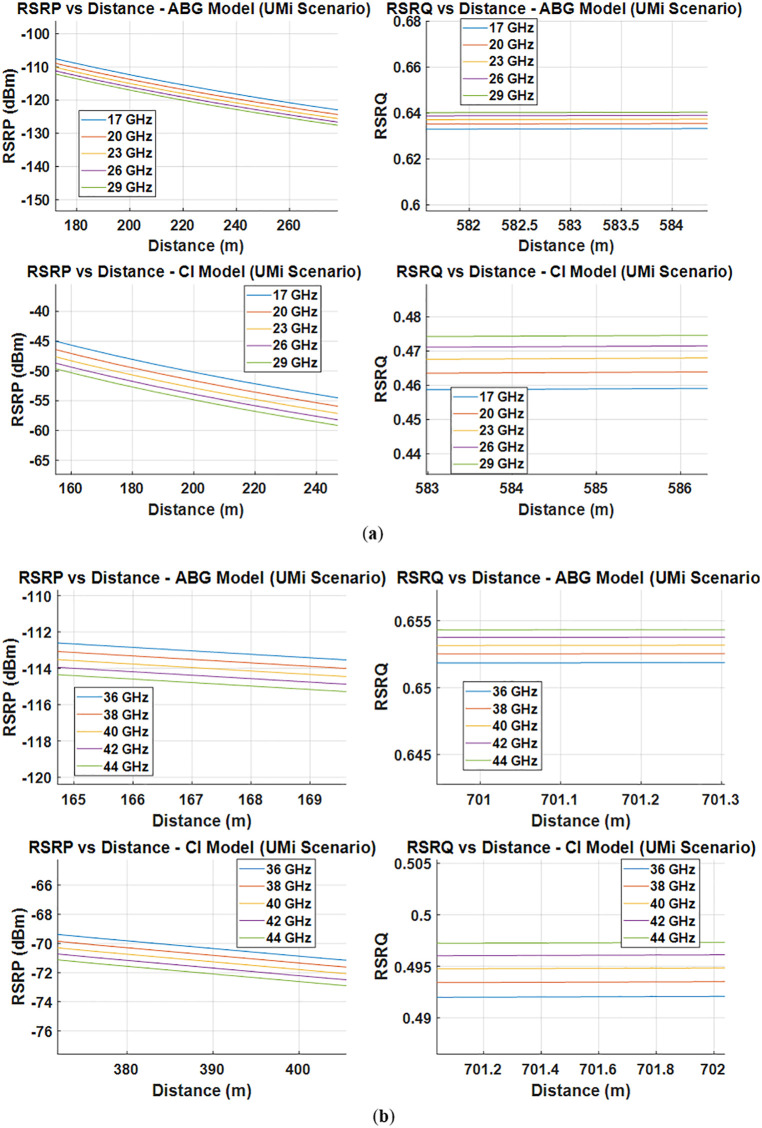
Comparison of RSRP and RSRQ vs Distance for ABG/CI Model in UMi Scenario for (a) Band-1 and (b) Band-2 frequencies.

The metric of received power versus distance is among the most important in 5G systems, as received power directly impacts signal quality and network coverage. As soon as the distance increases between the transmitter and receiver, the received power decreases, thus affecting the overall SNR and, therefore, leading to a loss of connection if the power falls below a certain threshold. This metric enables the planning and optimization of network deployment for application-specific communication over various distances. RSSI measures the total received power, including both the desired signal and any interference or noise, while RSRP measures only the power of the reference signals, which are part of the total power used for a more accurate assessment of network performance. Meanwhile, the received power versus distance focuses on the attenuation of signal power over a distance in general, helping assess network performance over distances bound by different environmental conditions. Fig 14(a)–14(b) shows the RSRP and RSRQ vs Distance for ABG and CI Models in UMi Scenario for Band-1 and Band-2. Let us consider the RSRP and RSRQ for ABG and CI models in the UMi scenario for Band-1; the first four subplots give a complete overview of the comparison. We already have from 3GPP specification that RSSI should be less than −120 dBm to have a meaningful connection from BS to MS. Considering the worst-case scenario of −120 dBm, Reference signal Received power and Reference Signal Received Quality have been plotted for Band-1 ABG/CI models in the top four subplots of Fig 6. For 250 meters distance, the CI model performs much better than the ABG model as it only needs −45 dBm to −50 dBm of power. Similarly, a Higher value of RSRQ is required for the ABG model in comparison to the CI model. Values of 0.62 and 0.64 are excellent but slightly on the higher side as compared to 0.47 over a CI model.

The second four subplots that follow depict the above trend for Band-2 as in Fig 14(b) in their entirety. It is known from 3GPP specifications that for any meaningful connection between BS-MS, the RSSI should be below −120 dBm. In the worst case of −116 dBm, RSRP and RSRQ for the Band-2 ABG/CI models are plotted in the bottom four subplots of Fig 6. Even for 390 meters, the CI model uses a value of −70 dBm, which is better than the ABG model as it requires −115 dBm to −118 dBm power for a mere distance of 170 meters from the BS. Similarly, ABG requires a higher RSRQ than the CI model. Values of RSRQ of 0.655 and 0.65 have been rated as excellent and yet a bit higher when compared to 0.49 for the CI model.

### 4.6 Complete path loss analysis in urban microenvironment

Urban micro path-loss models employ ABG and CI models, as they play a pivotal role in understanding signal attenuation in densely populated areas where buildings, vehicles, and other concrete structures significantly impede signal propagation. The ABG model has three variables that help account for most environmental factors and show elasticity across different scenarios. As seen from [28,25] this CI model is easier to apply under different situations. The distinction between Line-of-Sight (LoS) and Non-Line-of-Sight (NLoS) becomes particularly important in an urban environment because shadowing effects are caused by buildings and other large objects that block or reflect signals. In LOS conditions, the path a signal must take is clearer, whereas in NLoS conditions, multipath propagation occurs before the signals arrive at the receiver. This gets to be even worse when weather changes, like rain, fog, or snow, gets added to the signal strength and might affect propagation. Consequently, a proper understanding and correct modeling of these conditions using ABG and CI models is paramount for effective wireless communication network design in urban areas.

#### 4.6.1 The specific attenuation due to rain is given by [26].


$$\begin{array}{c} \gamma_{R}=k\cdot R^{\alpha }(dB/km) \end{array}$$ (5)

R is the rainfall rate in mm/hr, and k and α are frequency-dependent empirical coefficients from ITU-R P.838-3 (Table 1). For a terrestrial link, the relevant coefficients are:

It is observed from the figure that the fourfold increase in ***k*** from 21 GHz to 38 GHz is precisely drives the higher rain penalty at 38 GHz. Specific Attenuation

Applying Eq (5) for all three rainfall rates we get

At 21 GHz:


$$\begin{array}{c} \gamma_{R}(\text{10})=\;\text{0}.\text{0751}\times \;{\text{10}}^{\left\{\text{1}.\text{099}\right\}}=\;\text{0}.\text{94 }dB/km \end{array}$$

$$\begin{array}{c} \gamma_{R}(\text{15})=\;\text{0}.\text{0751}\times \;{\text{15}}^{\left\{\text{1}.\text{099}\right\}}=\;\text{1}.\text{47 }dB/km \end{array}$$

$$\begin{array}{c} \gamma_{R}(\text{25})=\;\text{0}.\text{0751}\times \;{\text{25}}^{\left\{\text{1}.\text{099}\right\}}=\;\text{2}.\text{58 }dB/km \end{array}$$

At 38 GHz:


$$\begin{array}{c} \gamma_{R}(\text{10})=\;\text{0}.\text{3081}\times \;{\text{10}}^{\left\{\text{0}.\text{9624}\right\}}=\;\text{2}.\text{825 }dB/km \end{array}$$

$$\begin{array}{c} \gamma_{R}(\text{15})=\;\text{0}.\text{3081}\times \;{\text{15}}^{\left\{\text{0}.\text{9624}\right\}}=\;\text{4}.\text{174 }dB/km \end{array}$$

$$\begin{array}{c} \gamma_{R}(\text{25})=\;\text{0}.\text{3081}\times \;{\text{25}}^{\left\{\text{0}.\text{9624}\right\}}=\;\text{6}.\text{824 }dB/km \end{array}$$

The 25 mm/hr case was included to reflect dense tropical and monsoon rainfall conditions relevant to the deployment scenarios considered in the paper.

By using [26], we can arrive at path attenuation, total rain attenuation over a link of distance d (km) is:


$$\begin{array}{c} A_{rain}\;=\gamma_{R}\times \;d\;dB \end{array}$$ (6)

At 25 mm/hr over 500 m, the rain penalty at 38 GHz is 3.31 dB more than 2.5 times the 21 GHz value of 1.28 dB. This difference is non-trivial in a 5G mmWave link budget and was accounted for accordingly. The ITU-R P.838−3 model is used solely for the rain attenuation component.

**Fig 15:**
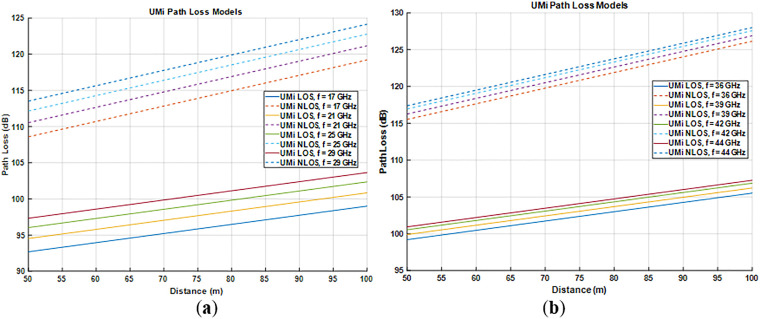
Path loss vs Distance Comparison in LoS and NLoS components for UMi Scenario over (a) Band-1 and (b) Band-2 frequencies.

Fig 15(a)–15(b) describes LoS and NLoS path loss analysis for Band-1 and Band-2. The performance of a dual-band mmWave antenna system in LoS/NLoS conditions under the UMi scenario differs due to the environment’s propagation characteristics. The path loss in LoS for Band-1 varies between 98–103 dB, where in NLoS conditions it goes up to 118–123 dB. Similarly, Band-2 has path loss in LoS of 106−108 dB, while for NLoS it is considerably higher at 126−128 dB. This variation in path loss between LoS and NLoS occurs because, in LoS conditions, there is a direct line of sight between the transmitter and receiver; in NLoS conditions, all other obstacles block the path; hence, signals have to travel a longer distance and are attenuated. The reason is that, under LOS conditions, the signal travels straight to the receiver, whereas under NLOS conditions, it encounters barriers such as buildings, trees, and other structures that can cause reflections, diffractions, and scatterings, thereby increasing path loss. Moreover, higher-frequency bands, such as Band-2, are more prone to attenuation due to their greater sensitivity to environmental obstacles, which explains their higher path loss compared to Band-1 under both LoS and NLoS conditions.

**Fig 16:**
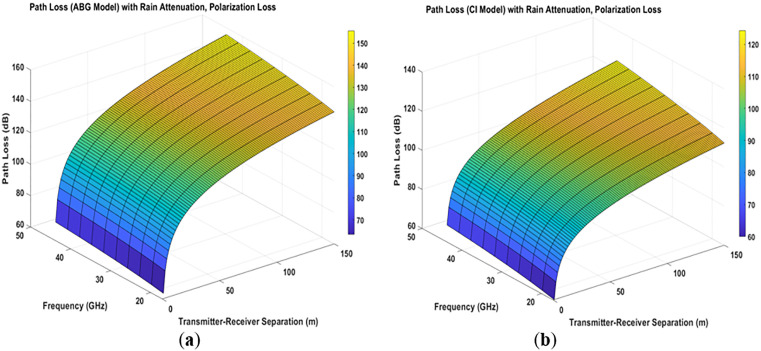
Path loss vs Distance vs Frequency for Band-1 and Band-2 frequencies in deep fade scenario, with rain attenuation and polarization loss for the designed antenna (a) ABG and (b) CI models.

Fig 16(a)–16(b) illustrates the path loss versus frequency for varying T-R separation over an ABG model for a designed antenna system. A path loss of 60dB is observed at the center frequency of 21 GHz for a distance of 10 meters in Band-1 and similarly, 66 dB path loss is observed for the center frequency at 39 GHz for Band-2, with the path loss increasing exponentially to 156 dB when the distance extends to 150 meters in a typical UMi environment. This highlights the importance of modeling path loss by considering both distance and frequency and emphasizes the need for specialized solutions to tackle challenges in different urban settings. The worst-case scenario has been used where Rain attenuation at a rate of (10–15 mm per hour with a shadow effect of 4 and polarization mismatch of up to 15 degrees is considered for this simulation [29,30]. Similarly, since the CI model’s construction is simple but highly efficient for path-loss modeling, particularly in smart city analysis. It is observed that with a frequency-dependent constant representing free-space path loss at a reference distance, usually one meter produces these results, A path loss of 56dB is observed at the center frequency of 21 GHz for a distance of 10 meters in Band-1 and similarly, 60 dB path loss is observed for the center frequency at 39 GHz for Band-2, with the path loss increasing exponentially to 128 dB when the distance extends to 150 meters in a typical UMi environment. Given its advantages in urban and sub-urban radio environments, this model is ideal for physical network deployments, especially for 5G and beyond, as indicated in references [25,28,29,30].

The antenna design now supports 5G frequencies around 21.6 GHz and 31.6 GHz, but because of its structure, it may be scaled for higher reaches such as those used for investigating 6G mmWave frequencies of 60–100 GHz. Studies [24] and [27] have pointed out that as frequencies increase, path loss and the overall characteristics of wave propagation become more complex, due to more absorption by the air, greater blockage sensitivity and additional diffraction losses. Even so, essential studies such as those in [28] and [25] demonstrate that mmWave and sub-THz bands are suitable for mobile applications when antenna arrays and beamforming are used to mitigate signal loss along each path.

## 5 Conclusions

The path-loss characteristics of a double-band mmWave antenna in the 17–27 GHz and 36–43 GHz frequency ranges are comprehensively examined, with particular focus on urban microcell and microcell environments. The proposed antenna has a lowered ground plane and a concentric arc-shaped radiating patch, specifically engineered for efficient performance within these bands to meet the demands of next-generation communication systems, with gains of 4.8 and 6.1 dBi at 26 GHz and 37 GHz, respectively. Based on thorough modeling and analysis, the antenna performs well in both frequency bands and is distinguished by its wide impedance bandwidth and low return loss (S11 < 10 dB). The proposed mmWave antenna’s gain makes it suitable for use in urban micro- and macro-cellular systems that demand high-capacity short-range communications. The antenna is fairly reliable in terms of its propagation loss, which varies as expected between the two frequency bands despite the different propagation mechanisms at 17–21 GHz and 36–44 GHz, as confirmed by the path-loss analysis conducted in practical urban scenarios.
